# Post Cardiac Surgery Acute Kidney Injury: A Woebegone Status Rejuvenated by the Novel Biomarkers

**DOI:** 10.5812/numonthly.19598

**Published:** 2014-07-05

**Authors:** Rajesh Jayaraman, Sham Sunder, Satyanand Sathi, Vijay Kumar Gupta, Neera Sharma, Prabhu Kanchi, Anurag Gupta, Sunil Kumar Daksh, Pranith Ram, Ashik Mohamed

**Affiliations:** 1 Department of Nephrology, Dr. Ram Manohar Lohia Hospital, Indraprastha University, New Delhi, India; 2 Department of Cardio Thoracic and Vascular Surgery, Dr. Ram Manohar Lohia Hospital, Indraprastha University, New Delhi, India; 3 Department of Biochemistry, Dr. Ram Manohar Lohia Hospital, Indraprastha University, New Delhi, India; 4 Prof. Brien Holden Eye Research Centre, LV Prasad Eye Institute, Hyderabad, India

**Keywords:** Acute Kidney Injury, NGAL Protein, Interleukin-18

## Abstract

**Background:**

Acute kidney injury (AKI) is common after cardiac surgery, the incidence varying between 7.7% and 28.1%. It significantly increases morbidity and mortality. Creatinine considerably delays the diagnosis with its own attended demerits. Novel urinary biomarkers are emerging which help in rapid diagnosis thus reducing the morbidity and mortality. Biomarkers of our study were neutrophil gelatinase-associated lipocalin (NGAL) and Interleukin-18 (IL-18).

**Objectives:**

To find out the incidence of AKI in post-cardiac surgery patients in our hospital, the ability of the two biomarkers in early diagnosis in predicting the severity of AKI based on RIFLE’s criteria and their ability to discriminate pre-renal from intrinsic AKI.

**Patients and Methods:**

One-hundred patients who underwent cardiac surgery were selected. Midstream urine samples were collected at 3 time intervals (baseline before surgery, 24 hours and 7 days after surgery). Biomarkers were measured by ELISA using BIORAD processors. Fractional excretion of sodium and urea were used to discriminate pre-renal from intrinsic AKI.

**Results:**

Out of 100 patients, 31 had AKI, 11 being pre-renal and 20 intrinsic AKI. Four patients required renal replacement therapy (12.9% among AKI cases and 4% in the overall study cohort). Four among 31 expired in intensive care unit. Identifiable risk factors for AKI included insulin requiring diabetes mellitus, chronic obstructive pulmonary disease, increased cardio-pulmonary bypass time, combined valvular surgery and coronary artery bypass grafting, employment of intra-aortic balloon counter pulsation, left main coronary artery occlusion and an ejection fraction of < 40%. NGAL was extremely sensitive (area under curve-0.96) in detecting intrinsic AKI at 24 hours followed by IL-18 ratio with an area under curve of 0.89. Creatinine at 24 hours was able to detect only 31.6% of intrinsic AKI. None of the pre-renal cases showed rise in the urinary biomarker levels. Patients with higher stages of AKI had higher levels of both biomarkers than those at lower stages.

**Conclusions:**

NGAL and IL-18 obviated the disadvantages of creatinine. They were efficient in early detection of AKI, in differentiating pre-renal from intrinsic AKI and in predicting the severity of AKI reliably in post-cardiac surgery patients.

## 1. Background

Acute kidney injury (AKI) is a common and serious complication of cardiothoracic surgery (1). Depending on the definition of AKI used, the incidence may be as low as 7.7% to as high as 28%, with 1% to 5% requiring renal replacement therapy (2-9). These criteria are still very much dependent on serum creatinine which has got multitude of demerits including its delayed rise in serum, inability to differentiate pre-renal from intrinsic AKI, being influenced by several non-renal factors and requirement of loss of at least 50% of renal function for it to rise in the serum. To obviate these disadvantages, several biomarkers are emerging in the new era of nephrology including neutrophil gelatinase-associated lipocalin (NGAL), interleukin-18 (IL-18), kidney injury molecule-1, α-glutathione-S-transferase and hepatocyte growth factor. NGAL is a 25-kD protein (10) that is covalently bound to gelatinase from neutrophils. NGAL is normally expressed at very low levels in several human tissues, such as the kidney, lungs, stomach, and colon. NGAL is an iron metabolism regulator at the level of renal tubular cells. The siderophore - iron associated-NGAL is taken up by the cell through megalin receptor apparatus. NGAL then concentrates in acidic endosomes that promotes the release and cytoplasmic accumulation of iron thus regulating iron dependent genes. Siderophore iron free NGAL (10) then captures the intracellular iron and transports it via an intracellular siderophore to the extracellular space. Depletion of NGAL leads to the depletion of intracellular iron pools resulting in the apoptosis of tubular cells and AKI. IL-18 is a pro-inflammatory cytokine (11) that plays a role in both the innate and acquired immune response. IL-18 plays an important role in host defenses against tumors and infections. Several studies in humans demonstrated the association of urine IL-18 with established tubular injury.

## 2. Objectives

To find out the incidence and risk factors of AKI in post cardiac surgery patients in our hospital, to assess the ability of urinary NGAL and IL-18 in the early detection of AKI, to differentiate of pre-renal from intrinsic AKI and to predict the severity of AKI as per the “Risk, Injury, Failure, Loss, End stage”, [RIFLE] criteria.

## 3. Patients and Methods

This is an open labeled non-randomized prospective observational study done for a period of 18 months from the time of enrolling the patients to the completion of the study. 100 patients were selected without any bias who got admitted in the cardiothoracic surgery department in Ram Manohar Lohia Hospital, New Delhi, India to undergo elective cardiac surgery between January 2012 and June 2013. Informed consent was obtained from all patients included in the study. Elective cardiac surgeries include coronary artery bypass grafting (CABG) both on and off pump, valve repairs, Mitral and aortic valve replacements and atrial and ventricular septal defect closures.

### 3.1. Inclusion and Exclusion Criteria

All the patients who took part in the study met the following inclusion criteria:

- Prospective patients above 18 years undergoing elective cardiac surgery.
- Patients who did not have any evidence of Pre-existing chronic kidney disease (CKD) as explained by normal kidney size and echogenic pattern in ultrasound kidney-ureter-bladder.
- Patients with normal pre-operative baseline blood urea and serum creatinine.
- Patients with no documented evidence of any AKI or recovery from AKI in the past.

All those who did not meet the above criteria were excluded from the study. Patients were evaluated at 3 definite pre-determined time points of their hospital stay including pre-operatively on the morning of the day of surgery, 24 hours after completion of the surgery, and 7 days after completion of the surgery.

Following were the patient’s characteristics and parameters estimated on the morning of the day of surgery, 24 hours and 7 days after surgery:

Demographic details, past medications, baseline creatinine in mg/dL, urine output in mL/h, 24 hours urinary creatinine in mg/dL, urinary NGAL in pg/mL, urinary NGAL adjusted to urinary creatinine in pg/mg of creatinine, urinary interleukin-18 in pg/mL and also adjusted to urinary creatinine in pg/mg of creatinine, and fractional excretion of sodium and urea (FE_Na_ and FE_Urea_). GFR was measured as per 4-variable modification of diet in renal diseases (MDRD) formula. Both FE_Na_ and FE_Urea_ were used to differentiate pre renal from intrinsic AKI. Biomarkers’ values were expressed in unadjusted forms and adjusted to urinary creatinine to correct for variations in urine flow. The pre-operative values were considered the baseline values of the patients. A detailed note of type and duration of surgery, intra operative complications if any and its treatment details, aortic cross clamp (ACC) time and cardio- pulmonary bypass (CPB) time if pump is employed was made. Fresh midstream urine samples were collected for the measurement of the biomarkers and urinary creatinine. Biomarkers were measured by ELISA using BIORAD ELISA processors employing the kits provided. Hycult biotech–HK 330 human NGAL ELISA kit was used for urinary NGAL measurement and IL-18 was measured using MBL human IL-18 ELISA kit. Urinary NGAL of < 60 pg/mL and IL-18 of < 257 pg/mL was considered as normal as per the standardization provided by the laboratory kits. Plasma and urine creatinine were measured by alkaline picrate reaction, blood urea by urease method and all the other routine investigations were done by “automated clinical chemistry analyzer”. Persons who did not develop AKI were taken as controls.

### 3.2. Statistical Analysis

Statistical analysis was performed using STATA 11.0 (Stata Corp LP, College Station, TX, USA). Shapiro-Wilk test was applied to test for normality of continuous variables. Parametric data were described as mean ± standard deviation. Median and inter-quartile range (IQR) were used to describe nonparametric data. Categorical data were analyzed using chi-square test. A P-value of < 0.05 was considered statistically significant. Area under curve (AUC) was utilized to determine at point of different parameters to distinguish between the subjects in AKI and normal (control) group.

## 4. Results

### 4.1. Observations

Out of 100 patients, 72 were male and 28 were female. Thirty-five patients underwent CABG alone, 53 underwent valve replacement alone, CABG and valve surgery included 5 patients and the remaining 7, other surgeries. There was no difference in length of ICU stay (P = 0.58, Mann-Whitney test) in those with AKI (median is 4 days, IQR 3 to 7 days) compared with those without AKI (median is 4 days, IQR 3.5 to 5 days). There was no difference in in-hospital mortality (P = 0.81, Chi-square test) in those with AKI (4/30) compared with those without AKI (8/69).

### 4.2. Acute Kidney Injury Its Incidence and Distribution

Thirty-one patients attained AKI as per the RIFLE’s criteria in different stages according to GFR and 69 did not and the latter formed the controls. Five patients were in the “risk” stage, 10 in the “injury” stage, 14 were in the “failure” stage and 2 in the “loss” stage of RIFLE. The baseline GFR of patients as per 4-variable MDRD formula who attained AKI in the study and who did not, are mentioned in the table below. The incidence is 33.3% in males 20.7% in females. Twenty-seven percent of patients were in the age group of 18-40 years, 33.3% in 41-60 age groups and the remaining were ≥ 61 years of age. Out of 31 cases, 4 required renal replacement therapy (12.9% among AKI cases and 4% in the overall study cohort) with one undergoing peritoneal dialysis and the other 3, hemodialysis. Four patients among these 31 expired in ICU. Among this mortality score, two were those who underwent dialysis and the other 2 did not. Table 1 shows the demographic profile of the patients in our study.

**Table 1.** Demographic Profile of Our Patients^a^

Characteristic	Number of AKI (n = 69)	Risk (n = 5)	Injury (n = 10)	Failure (n = 14)	Loss (n = 2)
**Age, y**					
Median	46	42	42	59	53
IQR	36-64	29-52	38-59	42-65	45-61
**Gender, %**					
Male	69.6	80	80	76.9	100
Female	30.4	20	20	23.1	0
**Baseline GFR (MDRD), mL/min, Mean ± SD**	112.71 ± 21.6	106.93 ± 7.75	118.51 ± 41.65	122.12 ± 25.99	89 ± 11.3
**Baseline Creatinine, mg/dL**					
Median	0.7	0.8	0.75	0.7	0.95
IQR	0.7-0.8	0.8-0.8	0.7-0.9	0.6-0.7	0.9-1
**Surgery type**					
CABG alone	24	2	2	6	1
Valve alone	39	1	5	7	1
CABG + Valve	1	1	2	1	0
Other	5	1	1	0	0
**CPB time, min, Mean ± SD**	100 ± 10.9	103.7 ± 24.7	116 ± 36.8	107.1 ± 38.3	119 ± 24
**Off-pump surgeryn**	19	2	1	4	0
**ICU**					
Median, d	4	3	3.5	6.5	30.5
IQR	3.5-5	3-4	3-5	4-7	27-34
Mortality, No	8	0	0	2	2

^a^ Abbreviations: AKI, acute kidney injury; GFR, glomerular filtration rate; MDRD, modification of diet in renal diseases; CPB, cardio-pulmonary bypass; ICU, intensive care unit; IQR, interquartile range.

The median baseline value of urine NGAL and NGAL to creatinine ratio (adjusted NGAL) was 33.19 pg/mL (IQR, 17.8 to 43.39) and 39.38 pg/mg (IQR, 26.07 to 47.84), respectively. The median baseline value of urine IL-18 and adjusted IL-18 was 97.99 pg/mL (IQR, 60.3 to 131) and 100.7 pg/mg (IQR, 66.78 to 141.34), respectively. The baseline urine NGAL in patients with AKI (median: 33.19 and IQR: 18.87 to 43.39) did not differ (P = 0.42, Mann-Whitney test) from controls (median: 33 and IQR: 15.3 to 42.22) and so did adjusted NGAL. [Median: 33.6 and IQR: 19.52 to 42.22 with AKI (P = 0.08, Mann-Whitney test) and median: 40.95 and IQR: 28.8 to 47.85 for controls]. The baseline urine IL-18 in patients with AKI (mean: 123.92 ± 87.61) did not differ (P = 0.21, Mann-Whitney test) from those who were controls (mean: 92.81 ± 43.54) and so with adjusted IL-18 (median: 124.07 and IQR: 49.6 to 180.67 with AKI and median: 98.44 and IQR: 72 to 129.4 for controls, P = 0.58, Mann- Whitney test).

### 4.3. Sub Classification of Acute Kidney Injury in Our Study

Besides the stage-wise classification of AKI based on RIFLE’S criteria, AKI was broadly classified into pre-renal and intrinsic AKI, based on clinical status, level of rise in creatinine, FE_Na_ and FE_Urea_ and improvement with hydration. Out of 31 AKI patients, 20 were found to have intrinsic and 11 pre-renal AKI, based on the above mentioned parameters.

### 4.4. Performance of the Novel Biomarkers and Creatinine at 24 hours After Surgery

**Figure 1.**
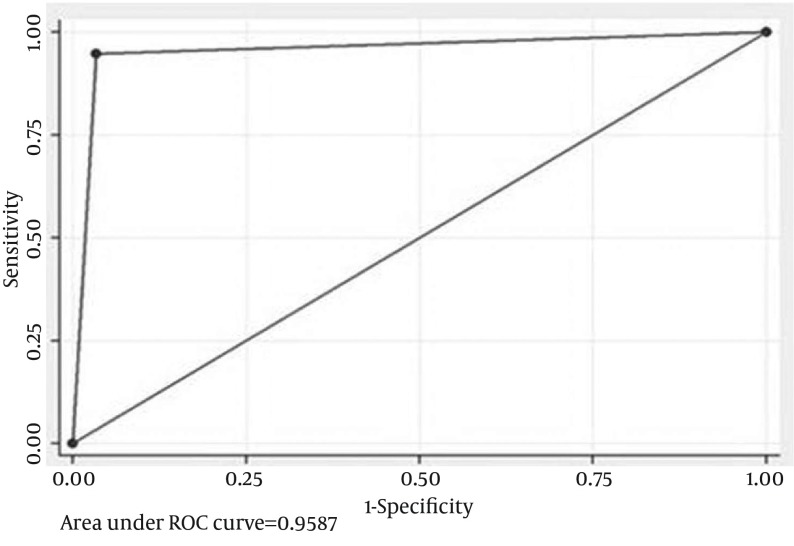
Receiver Operator Curve For Adjusted Neutrophil Gelatinase Associated Lipocalin at 24 Hours For Intrinsic Acute Kidney Injury Alone An AUC of 0.96 was observed.

**Figure 2.**
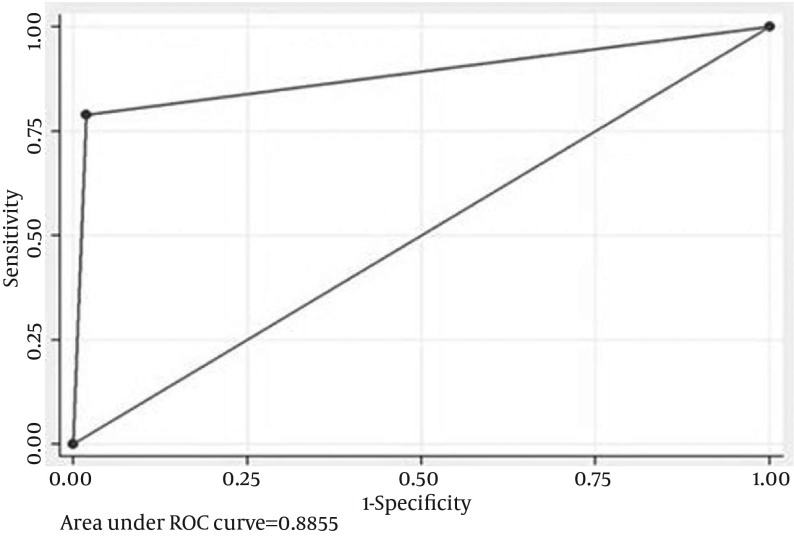
Receiver Operator Curve for IL-18 at 24 hours for Intrinsic Acute Kidney Injury Alone An AUC of 0.89 was observed.

In all 31 cases of AKI, NGAL > 60 pg/mL at 24 hours was seen in 72% (95% CI, 50.4% to 87.1%) (vs. GFR, P = 0.01, comparison of proportions). IL-18 > 250 pg/mL at 24 hours is seen in 48% (95% CI, 28.3% to 68.3%) (vs. GFR, P = 0.39, comparison of proportions). Twenty-four hours NGAL + IL-18 detected AKI in 72% (95% CI, 50.4% to 87.1%), and 24 hours NGAL + creatinine detected AKI in 80% (95% CI, 58.7% to 92.4%) (P = 0.51, comparison of proportions).Twenty-four hours NGAL alone detected AKI in 72% (95% CI, 50.4% to 87.1%), whereas 24 hours IL-18 alone detected AKI in 48% (95% CI, 28.3% to 68.3%) (P = 0.08, comparison of proportions). Neither the combination of NGAL and IL-18 nor NGAL + creatinine was found to be superior to NGAL alone in detecting AKI at 24 hours. The adjusted biomarker level to creatinine was not found to be superior to unadjusted value for NGAL in detecting AKI at 24 hours but was found to be superior for IL-18 NGAL and IL-18 being tubular markers are supposed to rise in the urine only when there is intrinsic tubular AKI and not in pre-renal AKI. Segregating these cases as 20 intrinsic and 11 pre-renal AKI and extrapolating the biomarker levels with these categories produced a more refined status for these biomarkers (Figures 1 and 2).

**Figure 3.**
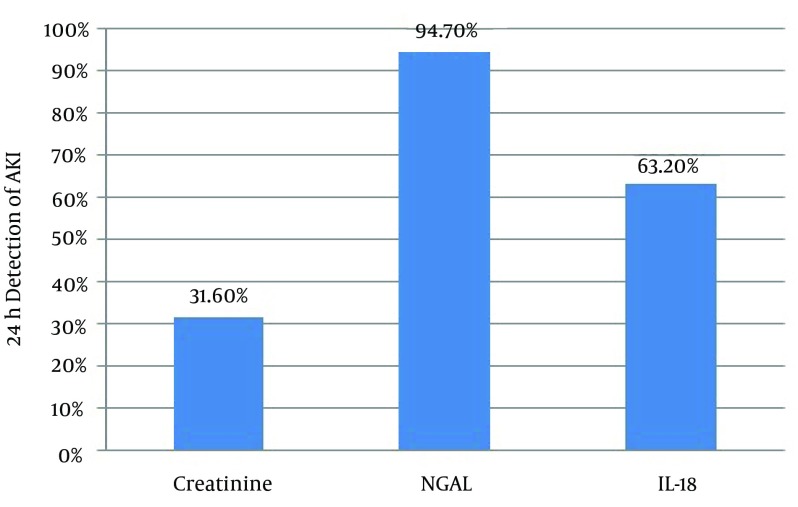
Histogram to Show the Ability of the Three Parameters to Detect Acute Kidney Injury at 24 hours

With this extrapolation, NGAL was found to be extremely sensitive (AUC-0.96) in detecting AKI at 24 hours followed by adjusted IL-18 with an AUC of 0 .89. Clearly these biomarkers were found to be superior to creatinine in early identification of AKI and in differentiating pre-renal from intrinsic tubular AKI (Figure 3).

A CPB time of > 100 minutes consistently increased the risk of AKI while ACC time did not have significant impact on AKI incidence. The median CPB time in AKI group (115 min, IQR-95 to 128 min) was significantly different from that of controls (84 min, IQR-78 to 94 min), (P = 0.03 by Mann-Whitney test).

### 4.5. Biomarkers in Predicting Increasing Severity of Acute Kidney Injury

**Figure 4.**
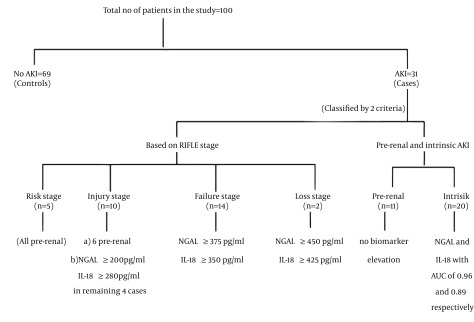
Algorithm Showing Overall Results and Outcomes in the Study

Patients at increasing stages of AKI had increased biomarker levels than the patients at lower stages. A 24 hours NGAL level of > 200 pg/mL was a consistent finding in patients with “injury” stage of AKI, while a value of 375 pg/mL and 450 pg/mL was observed in those with “failure” and “loss” stages respectively. The highest observed value for NGAL at 24 hours was 520 pg/mL in “loss” stage of “RIFLE”. IL-18 produced also produced similar results with a value of > 350 pg/mL being observed in AKI beyond “INJURY” stage (Figure 4).

## 5. Discussion

The incidence of postoperative AKI in patients undergoing cardiac surgery ranges from 7.7% to 28.1% (12) in different studies, probably in relation to the criteria adopted to define AKI. Thakar et al. (13), Bove et al. (14), Mangano et al. (6) in their large scale studies observed an incidence of 15.7%, 5.3% and 7.7% respectively in post-cardiac surgery patients. In our study, we observed an incidence of 31%. Conlon et al. (5) and Chertow et al. (4) in their large cohorts found an incidence of 1.7% and 1.1% respectively of AKI requiring dialysis. In our study, the incidence of AKI requiring dialysis was 4% in the overall cohort of patients and 12.9% among those who attained AKI. The established risk factors described in the development of post-cardiac surgery AKI include female gender, congestive cardiac failure, ejection fraction of < 40%, employment of intra-operative IABP, long CPB and ACC time, COPD, insulin requiring diabetes mellitus, left main coronary artery occlusion, prior cardiac surgery, pre-existing CKD and combined valvular surgery and CABG. Cleveland clinical scoring gives double scoring for pre-existing renal failure, congestive cardiac failure, emergency cardiac surgery, combined CABG and valvular surgery. In our study also, we encountered all the risk factors mentioned above including IABP (in one patient). In contrast to what is described in scoring systems, we observed a lesser incidence among females than in males (20.7 per 100 female cases and 33.3 per 100 male cases respectively). In a study by Koyner et al. (16), the diagnostic and prognostic utility of novel and traditional AKI biomarkers was evaluated during a prospective study of 123 adults undergoing cardiac surgery. Various biomarkers including NGAL were studied. The 6 hours ICU NGAL (AUC - 0.88; P < 0.001) best detected early stage 3 AKI. In a study done by Bennett et al. (17), 196 patients who underwent CABG were enrolled and evaluated for AKI. AKI developed in 99 patients in whom diagnosis due to creatinine was delayed up to 3 days after CPB. In contrast, mean urine NGAL levels increased 15-fold within 2 hours and by 25-fold at 4 and 6 hours after CPB. In our study, NGAL was found to be extremely sensitive in detecting AKI at 24 hours post cardiac surgery with an AUC of 0.96. Urine NGAL was found to be consistently increasing with increasing stages of AKI as per AKI network criteria. Similar to our study, both the biomarkers performed well with levels increasing consistently with increasing severity of AKI as per “RIFLE” criteria. In a study done by Singer et al. (18), among 145 patients, urinary NGAL levels effectively discriminated between intrinsic and pre-renal AKI (AUC- 0.87). An NGAL level over 104 pg/mL indicated intrinsic AKI (likelihood ratio 5.97), whereas an NGAL level of < 47 pg/mL made intrinsic AKI unlikely (likelihood ratio 0.2). In our study, none of our patients classified under pre-renal AKI had increased NGAL levels. In a study by Xin et al. (19), 33 patients undergoing CPB were classified as AKI (50% increase in serum creatinine within 48 h after CPB) and no AKI. Urine NGAL and IL-18 were increased in the AKI group at 2-4 hours postoperatively. Koyner et al. (20), used samples from the Translational Research Investigating Biomarker Endpoints in AKI study (TRIBE-AKI) of 1219 adults who underwent cardiac surgery and who had urine IL-18, urine NGAL and plasma NGAL estimated prior to surgery and for five post-operative days. Multivariate analysis revealed that the highest quintiles of urine IL-18 at six hours were strongly associated with risk of AKI (adjusted odds ratios of 6.8). In our study, urine IL-18 identified AKI at 24 hours with an AUC of 0.89 and reliably distinguished pre-renal from intrinsic AKI. AKI is common after cardiac surgery. We encountered an incidence of 31%. Novel urinary biomarkers of our study NGAL and IL-18 outsmarted creatinine. They were efficient in early detection of AKI, severity prediction and in discriminating pre-renal from intrinsic AKI reliably.
